# Sequencing of 10 complete *Curvularia* genomes isolated from rice in Burkina Faso

**DOI:** 10.1128/mra.00181-25

**Published:** 2026-09-01

**Authors:** Abdoul Kader Guigma, Nicolas Poncelet, Henri Adreit, Florian Charriat, Kadidia Koita, Issa Wonni, Didier Tharreau, Abalo Itolou Kassankogno

**Affiliations:** 1 Université Joseph KI ZERBO 107750, Ouagadougou, Burkina Faso; 2 CNRST/INERA, Laboratoire de Phytopathologie 317957 , Bobo-Dioulasso, Burkina Faso; 3 CIRAD, UMR PHIM 740764 , Montpellier, France; 4 PHIM, Université Montpellier, CIRAD, INRAE, IRD, Institut Agro Montpellier 27037, Montpellier, France; 5 ASTRE, CIRAD, INRAe, Université Montpellier 27037, Montpellier, France; 6 ENAFA de Matourkou, Bobo-Dioulasso, Burkina Faso; University of California Riverside, Riverside, California, USA

**Keywords:** fungi, brown leaf spot, *Curvularia*, complete genome, genomics DNA, molecular diagnostics

## Abstract

Rice is facing attacks from emerging diseases caused by fungi of the *Curvularia* genus. Twenty-one species have been isolated from rice. Of these, 8 species cause brown spot disease, and only 13 complete genome sequences are available. We produced 10 *Curvularia* genome sequences of strains isolated from diseased rice.

## ANNOUNCEMENT

Rice is the staple food for half of the world’s population and the second most widely grown cereal (1). Its production is constrained by several pathogens. Once considered minor, fungi of the genus *Curvularia* seem to be emerging threats. Eight *Curvularia* species have been reported to cause brown to black spots on leaves and seeds and rots on rice spikelets (2–4). To our knowledge, 21 *Curvularia* species have been isolated from rice, and only 13 genomes are available in databases. To identify and characterize the *Curvularia* species present on rice in West Africa, we produced additional complete genome sequences. Symptomatic rice seeds and leaves were collected in 2021 and 2022 in Burkina Faso (Table S1, https://doi.org/10.18167/DVN1/4WHJAJ) and incubated on moistened filter paper at 25°C, with 12/12 h light/dark cycle for 3 to 5 days. Single-spore isolations were performed. To produce mycelium for DNA extraction, 271 isolates were grown on potato dextrose agar (PDA) (39 g/L) covered with a cellophane disc for 7 days at 25°C. DNA was extracted from 60 mg of mycelium using cetyl trimethyl ammonium bromide (CTAB) (5) and used to produce Genotyping-By-Sequencing (GBS) libraries. A quality filter (Phred score threshold of 20) was applied using Atropos (6), and read quality was assessed using FastQC (7). GBS data permitted the assignment of 33% of isolates to a species and to group them. From these groups, 10 isolates were selected for full-genome sequencing. GAPDH and TEF gene sequences were extracted from assembled genomes by BLASTN (8) and aligned with the sequences of 80 strains, corresponding to 74 *Curvularia* species and representative of the phylogenies established by references 9, 10 to reconstruct phylogenies for each gene independently with the “one click” mode of the phylogeny.fr pipeline (11). Newly sequenced strains were assigned to *Curvularia pisi*, *C. soli*, *C. dactylocteniicola*, and *C. homomorpha* according to the species name of the closest reference strain, consistently in GAPDH and TEF phylogenetic trees.

Genomic DNA was extracted from mycelium produced in liquid PDA by the CTAB method (5). Libraries were prepared with Novogene NGS DNA Library Prep Set, and 150 bp paired-end sequences were obtained by Illumina Sequencing-By-Synthesis with NovaSeq 6000. Genomic DNA was fragmented by sonication to approximately 350 bp. After end repair, adenylation, adapter ligation, PCR enrichment, and AMPure XP purification, read quality was evaluated similarly as for the GBS data sets. ABySS v2.2.1 (12) was used to perform assembly with k-mer sizes from 20 to 90 in steps of 10 using default settings. The k-mer value giving the highest N50 was chosen for each strain. Only scaffolds longer than 500 bp were retained. Mating-type sequences were retrieved from assemblies using tBLASTN searches with AF129745.1, AF129746.1, KU749293, and KU749294. Genome length ranged from 31.4 to 33.8 Mb, scaffold number from 107 to 1,424, and guanine-cytosine (GC) content from 50 to 51% (Table 1). Genome completeness assessed using BUSCO V5.4.3 with the Pleosporales’ data set (13) showed that the assemblies recovered over 93% of the single-copy genes expected in the Pleosporales order. *Curvularia clavata* YC1106 genome, which is well assembled, has a completeness value (94.1%) practically the same to those of our genomes.

**TABLE 1:** General characteristics of the complete genomic sequences

	Sequencing					Assembly					BUSCO analysis				
Strain	Accession number	Putative species	Mating type	Number of reads	Coverage depth (×)	k-mer value	No. of scaffolds	Size (bp)	GC %	N50 (bp)	% of complete	% of complete and single copy	% of complete and duplicated	% of fragmented	% of missing
BF9368	CBCPGI010000000	*C. pisi*	MAT2	19,910,372	93.3	90	649	32,973,020	50	332,130	94.3	94.2	0.1	0.4	5.3
BF9418	CBCPGN010000000	*C. pisi*	MAT2	14,875,960	69.7	80	499	32,026,321	51	270,592	94.2	94.1	0.1	0.4	5.4
BF9436	CBCPGH010000000	*C. soli*	MAT2	15,567,580	72.9	80	107	31,887,855	51	1,119,627	94.1	93.9	0.2	0.3	5.6
BF9445	CBCPGJ010000000	*C. dactylocteniicola*	MAT2	15,989,204	74.9	80	893	33,739,466	51	282,021	94.4	94.1	0.3	0.3	5.3
BF9478	CBCPGG010000000	*C. soli*	MAT2	14,530,142	68.1	80	121	31,949,106	50	1,272,017	94.2	94.0	0.2	0.3	5.5
BF9509	CBCPGP010000000	*C. dactylocteniicola*	MAT2	17,749,258	83.1	70	1424	33,868,822	51	199,026	94.4	94.1	0.3	0.3	5.3
BF9539	CBCPGK010000000	*C. homomorpha*	MAT1	13,822,238	64.7	80	516	31,404,743	51	351,434	94.2	94.0	0.2	0.3	5.5
BF9580	CBCPGL010000000	*C. homomorpha*	MAT2	18,885,786	88.5	90	468	32,097,874	51	506,840	94.5	94.3	0.2	0.3	5.2
BF9594	CBCPGM010000000	*C. pisi*	MAT1	17,782,708	83.3	70	605	32,205,552	51	283,458	94.2	94.1	0.1	0.2	5.4
BF9599	CBCPGO010000000	*C. pisi*	MAT2	17,083,588	80.0	70	714	31,935,217	51	200,537	94.2	94.1	0.1	0.4	5.4

## Data Availability

Whole-genome sequences were deposited in ENA under the following assembly accession numbers: CBCPGI010000000, CBCPGN010000000, CBCPGH010000000, CBCPGJ010000000, CBCPGG010000000, CBCPGP010000000, CBCPGK010000000, CBCPGL010000000, CBCPGM010000000, and CBCPGO010000000. Sequences of TEF1 gene are available at https://www.ebi.ac.uk/ena/browser/view/OZ405193-OZ405202. Sequences of GAPDH gene are available at https://www.ebi.ac.uk/ena/browser/view/OZ405203-OZ405212.
